# Hyperlipidemia, statin use and dengue severity

**DOI:** 10.1038/s41598-018-35334-2

**Published:** 2018-11-21

**Authors:** Po Ying Chia, Htet Lin Htun, Wei Ping Ling, Yee Sin Leo, Tsin Wen Yeo, David Chien Boon Lye

**Affiliations:** 1 Communicable Diseases Centre, Institute of Infectious Disease and Epidemiology, Tan Tock Seng Hospital, Singapore, 308433 Singapore; 2 Department of Clinical Epidemiology, Office of Clinical Epidemiology, Analytics and Knowledge (OCEAN), Tan Tock Seng Hospital, Singapore, 308433 Singapore; 3 Lee Kong Chian School of Medicine, Nanyang Technological University, Singapore, 639798 Singapore; 4 Yong Loo Lin School of Medicine, National University of Singapore, Singapore, 119228 Singapore; 5 Saw Swee Hock School of Public Health, National University of Singapore, Singapore, 11754 Singapore

## Abstract

Multiple *in vitro* and mice model studies suggest statins may attenuate dengue severity. However, little is known about statin use and dengue severity in adult dengue patients with hyperlipidemia. We conducted a retrospective cohort study from 2004–2008 and 2012–2013 in Tan Tock Seng Hospital, Singapore on adult dengue patients with hyperlipidemia, comparing those with and without statin usage at hospitalization in terms of primary outcome of dengue hemorrhagic fever (DHF) or shock syndrome (DSS), and severe dengue (SD). Of 13,975 subjects screened, 257 dengue patients were included; 191 (74.3%) were statin users and 66 (25.7%) were non-users. Compared with non-users, statin use was not associated with decreased risk of DHF/DSS (adjusted risk ratio [aRR] = 0.66, 95%confidence interval [CI]: 0.41–1.08, P = 0.10) and SD (aRR = 1.43, 95%CI: 0.84–2.43, P = 0.19). Therefore, statin usage had minimal effect on dengue severity in our study population in Singapore.

## Introduction

Dengue is an arboviral disease of global importance, with increase in incidence attributed to globalization, changing climates, and geographical expansion^1^. It is caused by dengue virus (DENV) which has four serotypes. While majority of dengue patients recover uneventfully, a proportion will progress to severe illness which has no specific treatment available to date. The immune system plays an important role in dengue pathogenesis. It has been demonstrated that the peak in symptoms as well as progression to the critical phase of dengue occurs with viremia clearance and a rise in proinflammatory cytokines^2^. The pathogenesis of dengue hemorrhagic fever (DHF) or dengue shock syndrome (DSS) as defined in the World Health Organization (WHO) 1997 dengue guideline^3^, and severe dengue (SD) as defined in the WHO 2009 guideline^4^, involves vascular leakage which is associated with higher levels of various cytokines^5,6^.

Obesity, a non-communicable disease on the rise worldwide^7^, has been described as a risk factor for poorer outcomes in dengue^8^. Obesity is part of the metabolic syndrome which includes hyperlipidemia and is associated with an increased pro-inflammatory state^9^. Metabolic syndrome, obesity and hyperlipidemia are associated with various conditions such as nonalcoholic fatty liver disease, cardiovascular and cerebrovascular diseases, for which the treatment commonly includes statins. Statins are inhibitors of 3-hydroxy-3-methylglutaryl coenzyme A reductase, an enzyme necessary for cholesterol synthesis. With its pleiotropic effects, statins have been shown to reduce cytokines in various non-infective diseases^10–12^ and may potentially exert an immunomodulatory effect on the development of DHF/DSS and SD. Several retrospective and observational studies have documented better outcomes for patients already on statin therapy in various infectious such as pneumonia and bacteremia^13^. A few studies on *in vitro* and animal models for dengue infection also suggested that statin usage may improve disease outcomes^14–17^.

A common side effect of statins is a rise in transaminases, thus there is concern that continued use of statins in dengue infection can worsen clinical outcomes. Liver complications with raised transaminases of >1000 IU/L is one criterion for SD in the WHO 2009 dengue guideline^4^. Consequently, most clinicians discontinue statins upon suspicion of dengue infection and restart them only after recovery. A recent randomized controlled trial of Vietnamese adult dengue patients compared lovastatin versus placebo and did not find an increase in adverse events. However, the trial recruited relatively young adults who were unlikely to have comorbidities and was inadequately powered for dengue severity which was part of its secondary outcomes^18^. Thus, currently it is still unclear if preceding statin use influences the risk of dengue severity in adults with hyperlipidemia.

We embarked on this retrospective cohort study to investigate the association between preceding statin usage and dengue severity in adults with known hyperlipidemia presenting with dengue. We hypothesized that patients on statins had a decreased risk of plasma leakage with no increase risk in liver inflammation.

## Results

A total of 13,975 subjects were screened and 257 dengue patients with history of hyperlipidemia were included, including 191 (74.3%) statin users and 66 (25.7%) non-users. Of the 191 statin users, majority were on simvastatin, at 132 users (69.1%), followed by lovastatin at 33 users (17.3%), atorvastatin at 16 users (8.4%), rosuvastatin at 8 users (4.2%) and pravastatin at 2 users (1.0%). The mean age was 61.6 years with standard deviation (SD) of 12.0 years for users and 60.5 years with SD of 11.9 years for non-users, with 93 (48.7%) and 33 (50.0%) males respectively (Table 1). Statin users were significantly more likely to be overweight or obese, have a higher CCI index, and a history of hypertension in keeping with metabolic syndrome compared with non-users. A higher proportion of users were observed to have chronic liver disease (P = 0.07), but not other comorbidities (Supplementary Table 1). Similarly, users had more concurrent medications, and a statistically significant higher proportion was also on sulfonylurea, metformin, insulin, antiplatelet agents, ACEI and ARB.

**Table 1:** Demographic and Clinical Characteristics, and Dengue Severity between Statin Non-users and Users.

Characteristics	Total (n = 257)	Statin non-users (n = 66)	Statin users (n = 191)	P
**Demographics**				
Age ― years				
Mean ± SD	61.3 ± 11.9	60.5 ± 11.9	61.6 ± 12.0	0.53
Range	29–97	40–92	29–97	
Gender				
Male	126 (49.0)	33 (50.0)	93 (48.7)	0.86
Female	131 (51.0)	33 (50.0)	98 (51.3)	
**Health Risk Factors**				
Body mass index (BMI)				
≤24.99	113 (44.0)	28 (42.4)	85 (44.5)	0.02^†^
≥25–29.99	86 (33.5)	18 (27.3)	68 (35.6)	
≥30	41 (15.9)	10 (15.2)	31 (16.2)	
Missing	17 (6.6)	10 (15.1)	7 (3.7)	
Current smokers	24 (9.3)	7 (10.6)	17 (8.9)	0.68
**Comorbidities**				
Charlson’s comorbidity index (CCI) [linear]				
Median (IQR)	1 (1–3)	1 (0–3)	1 (1–4)	<0.01^‡^
Charlson’s comorbidity index (CCI) [categorical]				
0	50 (19.5)	25 (37.9)	25 (13.1)	<0.001
1	90 (35.0)	19 (28.8)	71 (37.2)	
2	32 (12.4)	4 (6.1)	28 (14.7)	
3	21 (8.2)	2 (3.0)	19 (9.9)	
≥4	64 (24.9)	16 (24.2)	48 (25.1)	
Other comorbidities				
Hypertension	197 (76.7)	40 (60.6)	157 (82.2)	<0.001
Cerebrovascular disease	33 (12.8)	6 (9.1)	27 (14.1)	0.29
Myocardial infarction	21 (8.2)	2 (3.0)	19 (10.0)	0.08
Congestive heart failure	10 (3.9)	1 (1.5)	9 (4.7)	0.46^†^
Atrial fibrillation	11 (4.3)	1 (1.5)	10 (5.2)	0.030^†^
Immunocompromised status	17 (6.6)	3 (4.6)	14 (7.3)	0.57^†^
Allergy	75 (29.2)	23 (34.9)	52 (27.2)	0.24^†^
Chronic liver disease	10 (3.9)	0 (0.0)	10 (5.2)	0.07^†^
**Concurrent medications**				
Other lipid-lowering agents*	17 (6.6)	6 (9.1)	11 (5.8)	0.39^†^
Sulfonylurea	87 (33.9)	10 (15.2)	77 (40.3)	<0.001
Metformin	120 (46.7)	9 (13.6)	111 (58.1)	<0.001
Insulin	26 (10.1)	2 (3.0)	24 (12.6)	0.03
Anti-platelet agents^#^	68 (26.5)	3 (4.6)	65 (34.0)	<0.001
ACE inhibitors	83 (32.3)	10 (15.2)	73 (38.2)	<0.01
Angiotensin-receptor blockers	41 (15.9)	4 (6.1)	37 (19.4)	00.01
Anti-arrhythmic drugs	6 (2.3)	0 (0.0)	6 (3.1)	0.34^†^
**Laboratory values**				
Total cholesterol^¥^ – mmol/L				
<5.2	130 (50.6)	19 (28.8)	111 (58.1)	<0.01
5.2–6.1	26 (10.1)	9 (13.6)	17 (8.9)	
>6.1	23 (8.9)	9 (13.6)	14 (7.3)	
missing	78 (30.4)	29 (43.9)	49 (25.7)	
LDL cholesterol^¥^ – mmol/L				
<2.6	102 (39.7)	13 (19.7)	89 (46.6)	<0.001
2.6–3.3	35 (13.6)	6 (9.1)	29 (15.2)	
3.4–4.1	26 (10.1)	11 (16.7)	15 (7.8)	
>4.2	16 (6.2)	6 (9.1)	10 (5.2)	
missing	78 (30.4)	30 (45.4)	48 (25.1)	
HDL cholesterol^¥^ – mmol/L				
>1.53	42 (16.3)	8 (12.1)	34 (17.8)	<0.01
1.03–1.53	75 (29.2)	12 (18.2)	63 (33.0)	
<1.03	63 (24.5)	16 (24.2)	47 (24.6)	
missing	77 (30.0)	30 (45.5)	47 (24.6)	
Triglyceride^¥^ – mmol/L				
<1.69	117 (45.5)	21 (31.8)	96 (50.3)	0.01
1.69–2.25	30 (11.7)	5 (7.6)	25 (13.1)	
>2.26	34 (13.2)	11 (16.7)	23 (12.0)	
missing	76 (29.6)	29 (43.9)	47 (24.6)	
Alanine aminotransferase (ALT)^§^ – IU/L				
Normal range	116 (45.1)	28 (42.4)	88 (46.1)	0.17^†^
>ULN	129 (50.2)	32 (48.5)	97 (50.8)	
missing	12 (4.7)	6 (9.1)	6 (3.1)	
ALT ≥ 500 IU/L	10 (3.9)	1 (1.5)	9 (4.7)	0.46^†^
ALT ≥ 1000 IU/L	6 (2.3)	1 (1.5)	5 (2.6)	1.00^†^
Aspartate aminotransferase (AST)^₸^ – IU/L				
Normal range	45 (17.5)	9 (13.6)	36 (18.8)	0.13^†^
>ULN	197 (76.7)	50 (75.8)	147 (77.0)	
missing	15 (5.8)	7 (10.6)	8 (4.2)	
AST ≥ 500 IU/L	16 (6.2)	3 (4.6)	13 (6.8)	0.77^†^
AST ≥ 1000 IU/L	11 (4.3)	1 (1.5)	10 (5.2)	0.30^†^
**Epidemic Year**				
Year of presentation				
2004, 2005, 2006, 2013 (DENV-1)	180 (70.0)	44 (66.7)	136 (71.2)	0.49
2007, 2008, 2012 (DENV-2)	77 (30.0)	22 (33.3)	55 (28.8)	
**Dengue severity**				
WHO 1997 dengue classification				
Dengue hemorrhagic fever/Dengue shock syndrome	80 (31.1)	24 (36.4)	56 (29.3)	0.29
▪ Hemorrhagic manifestations	114 (44.4)	28 (42.4)	86 (45.0)	0.71
▪ Plasma leakage	127 (49.4)	35 (53.0)	92 (48.2)	0.5
WHO 2009 dengue classification				
Severe dengue	81 (31.5)	17 (25.8)	64 (33.5)	0.24
▪ Severe bleeding	34 (13.2)	9 (13.6)	25 (13.1)	0.91
▪ Severe plasma leakage	89 (34.6)	16 (24.2)	73 (38.2)	0.04
▪ Severe organ impairment	41 (16.0)	8 (12.1)	33 (17.3)	0.32

Values are no. (%) unless stated otherwise.

^§^The ULN for ALT is 63 IU/L for males and 54 IU/L for females. Highest values within hospitalization were used for this analysis.

^₸^The ULN for AST is 41 IU/L. Highest values within hospitalization were used for this analysis.

^†^Fischer’s exact test.

^‡^Mann-Whitney U test.

*Other lipid-lowering agents include fenofibrate, gemfibrozil and ezetimibe.

^#^Anti-platelet agents include aspirin, clopidogrel and ticlopidine.

^¥^Lipid profile up to 1 year prior to admission were screened and the most recent record for lipid profile was chosen.

Statistically significant differences were noted in proportion of total cholesterol, LDL cholesterol, HDL cholesterol and triglyceride between users and non-users, with non-users having higher values. There was no statistically significant difference in ALT or AST levels at the end of hospitalization between the two groups, with a median ALT of 65IU/L (interquartile range [IQR]: 34–117 IU/L) in users and 68IU/L (IQR: 31–116 IU/L) in non-users (P = 0.69), and median AST of 94IU/L (IQR: 53–163 IU/L) in users and non-users 104 IU/L (IQR: 50–174 IU/L) (P = 0.78). There was no significant difference in proportion of patients with ALT ≥ 1000IU/L and AST ≥ 1000IU/L (Table 1).

Statin users were more likely to develop tachycardia (91 [47.6%] statin users versus 13 [19.7%] non-users, P = < 0.001) and fluid accumulation (41 [21.5%] statin users versus 4 [6.1%] non-users, P = 0.01) than non-users (Supplementary Table 1). There was however no difference between platelet nadir between the two groups (Supplementary Table 1). With regards to disease severity, there was no significant difference in occurrence of overall DHF/DSS (56 [29.3%] statin users versus 24 [36.4%] non-users, P = 0.29) and SD (64 [33.5%] statin users versus 17 [25.8%] non-users, P = 0.24) (Table 1). Interestingly, a higher proportion of users (33.5%) were more likely to have severe plasma leakage as defined by WHO 2009 guidelines, compared to non-users (25.8%) (P = 0.04).

Compared with non-users, usage of statins prior to admission was not associated with a reduced risk of DHF/DSS on multivariate analysis, after adjusting for potential confounders including age, gender, year of presentation, BMI, CCI, hypertension, and other medications including antiplatelet drugs, ARB, ACEI, insulin, metformin and sulfonylurea. There was no statistically significant risk reduction for both DHF/DSS (aRR = 0.73 95%CI 0.46–1.16, P = 0.18) and SD (aRR 1.52, 95%CI: 0.91–2.53, P = 0.11) (Table 2). There was also no significant difference in clinical outcomes, including length of stay, ICU admission and mortality between the two groups (Supplementary Table 1).

**Table 2:** Risk ratio for association between statin exposure and dengue severity.

Exposure	No. of patients	No. (%) of severe dengue manifestations	Crude RR (95% CI)	P	Adjusted RR^a^ (95% CI)	P	Adjusted RR^b^ (95% CI)	P
**Dengue hemorrhagic fever and shock syndrome:**								
Non-users	66	24 (36.4)	1.00		1.00		1.00	
Users	191	56 (29.3)	0.81 (0.55–1.19)	0.30	0.81 (0.55–2.2)	0.30	0.73 (0.46–1.16)	0.18
**Severe dengue:**								
Non-users	66	17 (25.8)	1.00		1.00		1.00	
Users	191	64 (33.5)	1.30 (0.82–2.05)	0.26	1.28 (0.82–2.01)	0.28	1.52 (0.91–2.53)	0.11

^a^Adjusted for age, gender.

^b^Adjusted for age, gender, year of presentation, BMI category, Charlson’s comorbidity index category, hypertension, myocardial infarction, chronic liver disease, concurrent medications usage including anti-platelet drugs, ARB, ACEI, insulin, metformin, sulfonylurea, and lipid panel including LDL-C, HDL-C, triglycerides and total cholesterol as categorical variables.

## Discussion

In this retrospective cohort study of laboratory confirmed adult dengue patients with hyperlipidemia, use of statins prior to hospital presentation was not associated with lower risk of DHF/DSS and SD. There was also no evidence of increased liver function abnormalities between dengue patients with or without statin use. A significantly higher proportion of users were more likely to develop severe plasma leakage as defined by WHO 2009 guidelines, compared to non-users. In our study, users of statin compared to non-users, had lower values of total cholesterol, LDL cholesterol, HDL cholesterol and triglyceride which may account for our findings. A similar phenomenon has also been reported in children, wherein lower levels of total cholesterol, LDL cholesterol and HDL cholesterol were observed in children with severe dengue when compared with children with mild dengue^19,20^, suggesting that cholesterol plays an important role in dengue pathophysiology, which needs to be further delineated.

DENV can infect a wide range of cells ranging from monocytes to endothelial cells *in vitro*^21^. T cells are activated, resulting in production of inflammatory cytokines and chemokines^2^. Statins may potentially exert immunomodulatory effects on the innate and adaptive immune response, as well as the vascular system. Briefly, higher levels of certain cytokines and chemokines such as interleukin (IL) 2, IL4, IL6, IL8, IL10, interferon (IFN)–γ, IFN-γ–induced protein 10 (IP-10), matrix metalloproteinases (MMP) 2 and MMP9 are associated with poorer prognosis^5,22–24^. *In vitro* non-dengue studies using murine cells, human bronchial epithelial cells and human monocytes showed a decrease in production of IL2, IL4, IL6, IL8, IFN- γ, MMP2 and MMP6 in the presence of statins^25–27^. In patients with various underlying medical conditions, ranging from autoimmune disease to cardiac disease, use of statins resulted in decreased IL6, IL10, IP10, MMP2 and MMP6^10–12^. Inhibition of cholesterol synthesis by statins has been shown to directly reduce virion assembly and regulate DENV replication *in vitro*^16,17^, and this may result in shortened viremia and better outcomes. In murine models however, use of statins had increased viremia and yet improved survival thus there is conflicting data on the impact statins has on viremia^15^.

Currently, only one randomized controlled trial on the use of statin in 300 dengue patients has been conducted^18^, comparing lovastatin versus placebo. While the trial demonstrated that concurrent use of lovastatin was safe with ongoing dengue infection as its primary outcome, there was no statistically significant difference in outcomes either in duration of fever or viremia, or disease severity. Only 3 patients out of 300 developed DHF/DSS or SD; thus, the study was inadequately powered to study statin effect on disease severity in dengue. There is also no other literature on the use of statins in other flaviviruses or arboviruses. While there is literature on influenza and statin use, the differences between the viruses and diseases precludes extrapolation.

Interestingly, our data showed a significant increased plasma leakage by the WHO 2009 definition in users versus non-users, however this was not seen when the definition in the WHO 1997 guideline was used. The difference in outcome analysis was driven by the inclusion of hypoproteinemia in the 1997 definition and a higher proportion of non-users having hypoproteinemia. Nevertheless, overall our data suggests that discontinuation of statins may not be necessary at the onset of dengue infection, and clinicians may adopt a watchful approach of continuing statins, supporting results shown by the Vietnam group^18^. Discontinuation of statins may not be harmless even for short periods of time^28^. Abrupt statin withdrawal was shown to cause impaired endothelial function via nitric oxide release and higher C-reactive protein levels resulting in a pro-inflammatory state^28^. In high risk patients with angina, discontinuation of statins resulted in a three-fold increase in cardiac risk compared with patients who continued statin treatment and 1.7 times increase in cardiac risk compared with patients who were statin-naïve^29^. With an ageing population compounded by a rise in obesity and associated comorbidities in Singapore, it is not uncommon that patients can present with dengue on a background of multiple medical problems that may pose a therapeutic challenge.

Our study has several strengths. Firstly, we used DHF/DSS and SD in our analysis as these classifications are internationally established as clinically relevant endpoints of dengue severity. Secondly, the dengue outcome documented has minimal risk of outcome information bias as a standardized dengue care path was used by all clinicians to assess dengue patients in our hospital. Thirdly, risk of statin usage misclassification was low because statin usage was ascertained from medication prescription database as well as medication reconciliation records performed on hospital admission by a hospital pharmacist. Lastly, clinical data was collected by two trained research assistants, minimizing error in data extraction and standardizing data collection. Data audit was conducted to ensure data accuracy.

Limitations of our study include the relatively small study population, the lack of evaluation of patient’s compliance to statin use, and missing lipid profiles for about 30% of our study population. Although some patients may not be compliant with medications after the onset of dengue symptoms prior to hospital admission, these patients were still nonetheless recorded as users. This would make it more challenging to determine the effects of the duration and dosage of statin and its potential immuno-modulatory effects in dengue infection. Nevertheless, in the analysis of patients with available lipid profile, patients in the statin arm were more likely to have optimal LDL-C and triglyceride levels. It is thus reasonable to assume that therapeutic concentrations for lipid-lowering effects were achieved in users. Although it is unclear if this is the same dosage required for an immuno-modulatory effect, prior observational reports on improved clinical outcomes in sepsis were with the routine dosages of statins^30,31^. Other limitations include the lack of data to adjust for the effect of different dengue serotypes on disease severity. The potential effect of prior dengue infection on disease severity also could not be adjusted as serostatus was not available in clinical practice. There is a risk of channeling bias, namely statin prescription is dependent on patient’s characteristics such as age, gender, ethnicity and comorbidities, but this was mitigated by adjustment in multivariable analysis. Finally, the study was not sufficiently powered to examine the interactions and effects statin might exert as a whole and differentially on different subgroups of patients.

## Conclusion

In adult dengue patients, statin usage at hospital admission did not reduce dengue severity in subjects with hyperlipidemia presenting with dengue. However, prior use of statins is not a risk factor for increased liver inflammation and supports the safety of continuing statins in patients with dengue.

## Methods

We conducted a retrospective cohort study using patients from previous studies with similar study designs from 2004–2008 and 2012–2013 in Tan Tock Seng Hospital, a 1600-bed teaching hospital in Singapore. Data collected included baseline demographic data (age, gender and ethnicity), year of illness, comorbidities (including Charlson’s comorbidity index (CCI), hypertension and other immunocompromising status), weight and height, smoking, daily symptoms and signs of illness, laboratory investigations (full blood count, renal panel, liver panel and lipid profile), radiology, and prescribed medications from hospital admission to discharge. Lipid profile up to 1 year prior to admission were screened and included total cholesterol, low-density lipoprotein cholesterol (LDL-C), high-density lipoprotein cholesterol (HDL-C) and triglyceride. The most recent record for lipid profile was documented. Clinical data from hospital admission to discharge date for inpatients, or to the end of acute follow-up for outpatients were documented using a standardized dengue care path for all dengue patients and data were extracted by trained research assistants. We selected a cohort of laboratory-confirmed hospitalized dengue patients with hyperlipidemia. Laboratory confirmation of dengue was based on positive non-structural protein 1 (SD BIOLINE Dengue Duo Standard Diagnostics, Republic of Korea) or polymerase chain reaction (PCR) results^32^. Study exposure was current use of any statin and subjects were classified as ‘users’ if they were on statin treatment at the time of hospital admission for dengue. The study outcome was dengue categorized as DHF/DSS or SD as per WHO 1997 and 2009 guidelines respectively^3,4^. Briefly, DHF requires the presence of fever, thrombocytopenia, any bleeding and evidence of plasma leakage. Patients with DHF who experienced shock were classified as DSS^3^. Patients with severe plasma leakage (respiratory compromise or shock), severe bleeding and severe organ impairment (renal impairment, aspartate aminotransaminase (AST) or alanine aminotransaminase (ALT) > 1000 units/L, encephalopathy, myocarditis) were classified as SD^4^.

### Potential confounders

Covariates likely to be associated with dengue severity and likelihood of receiving statin treatment were identified. Potential confounders include age, gender, comorbidities, obesity (body mass index (BMI) ≥ 30 kg/m^2^), smoking, and concurrent medications (sulfonylureas, metformin, insulin, anti-platelet agents, angiotensin-converting-enzyme inhibitors [ACEI], angiotensin-receptor blockers [ARB], anti-arrhythmic drugs and other lipid-lowering agents). DENV serotype is a known risk factor for disease severity^33^. As majority of the retrospective cohorts did not have PCR performed, the year of presentation was used as a surrogate marker for dengue serotype, postulating the serotype will be the predominant circulating dengue virus serotype of the epidemic year. Based on the Singapore communicable disease surveillance data from Ministry of Health Singapore, DENV-1 was the predominant serotype during 2004–2006 and 2013(61–82% of all circulating serotypes), and DENV-2 was the predominant serotype during 2007–2008 and 2012 (66–87%)^34^.

### Statistical methods

In order to investigate the association between statin use and severe dengue manifestations, we used univariate and multivariate Poisson regression with robust error variance^35^ to estimate crude and adjusted risk ratio (cRR and aRR) respectively with 95% confidence interval (CI). Multivariable models were built by adding a *priori* defined clinically important variables including age, gender, chronic liver disease, year of presentation and variables with P < 0.20 between the two groups. The best fit model was compared against nested model with Akaike information criterion (AIC)^36^. All statistical analyses were carried out with Stata 13.1 (College Station, TX: StataCorp LP). Statistical significance was set at P < 0.05 and all reported P values were two-tailed. The datasets used and/or analysed during the current study are available from the corresponding author on reasonable request.

### Ethics consideration

The study was approved by Singapore National Healthcare Group Domain Specific Review Board (NHG DSRB – 2015/01053) with waiver of informed consent. All subject identifiers were anonymized for analysis.

## Electronic supplementary material

Supplementary Table 1

## Data Availability

All data supporting the findings of this study are available within the article or from the corresponding author upon reasonable request.
