# Supplementary material for: Hyperlipidemia, statin use and dengue severity
Source: Sci Rep. 2018 Nov 21;8:17147. doi: 10.1038/s41598-018-35334-2 (PMC6249290; doi:10.1038/s41598-018-35334-2)
Supplement: Supplementary file 1 — Supplementary Table 1 [file 41598_2018_35334_MOESM1_ESM.docx]

**Hyperlipidemia, statin use and dengue severity**

Po Ying Chia^1^, Htet Lin Htun^2^, Wei Ping Ling^1^, Yee Sin Leo^1,3,4,5^, Tsin Wen Yeo^1,3^, and David Chien Boon Lye^1,3,4 *^

1. Communicable Diseases Centre, Institute of Infectious Disease and Epidemiology, Tan Tock Seng Hospital, Singapore 308433
2. Department of Clinical Epidemiology, Office of Clinical Epidemiology, Analytics and Knowledge (OCEAN), Tan Tock Seng Hospital, Singapore 308433
3. Lee Kong Chian School of Medicine, Nanyang Technological University, Singapore 639798
4. Yong Loo Lin School of Medicine, National University of Singapore, Singapore 119228
5. Saw Swee Hock School of Public Health, National University of Singapore, Singapore 11754

**Supplementary Table 1.** Clinical characteristics of statin users and non-users

| **Characteristics** | **Total**  **(n = 257)** | **Statin non-users**  **(n = 66)** | **Statin users**  **(n = 191)** | **P** |
| --- | --- | --- | --- | --- |
| **Comorbidities** |  |  |  |  |
| Peripheral vascular disease | 3 (1.2) | 0 (0.0) | 3 (1.6) | .57 |
| Peptic ulcer disease | 9 (3.5) | 5 (7.6) | 4 (2.1) | .05 |
| Hemiplegia | 15 (5.8) | 2 (3.0) | 13 (6.8) | .37 |
| Chronic pulmonary disease | 22 (8.6) | 4 (6.1) | 18 (9.4) | .40 |
| Renal disease | 46 (17.9) | 11 (16.7) | 35 (18.3) | .76 |
| **Day of fever on presentation**  Median (IQR)  Range | 4 (3 – 5)  1 – 16 | 4 (3 – 4)  1 – 9 | 4 (3 – 5)  1 – 16 | .49 |
| **Clinical manifestations** | | | | |
| Fever | 257 (100.0) | 66 (100.0) | 191 (100.0) | – |
| Aches and pains | 206 (80.2) | 55 (83.3) | 151 (79.1) | .45 |
| Rash | 122 (47.5) | 29 (43.9) | 93 (48.7) | .51 |
| Nausea or vomiting | 162 (63.0) | 44 (66.7) | 118 (61.8) | .48 |
| Tachycardia (Pulse rate > 100/min) | 104 (40.5) | 13 (19.7) | 91 (47.6) | < .001 |
| Hypotension  (Systolic blood pressure ≤ 90mmHg) | 40 (15.6) | 6 (9.1) | 34 (17.8) | .09 |
| Narrow pulse pressure | 5 (1.9) | 1 (1.5) | 4 (2.1) | 1.00 |
| **Warning Signs** | | | | |
| Abdominal Pain | 84 (32.7) | 19 (28.8) | 65 (34.0) | .43 |
| Persistent vomiting | 31 (12.1) | 9 (13.6) | 22 (11.5) | .65 |
| Fluid accumulation | 45 (17.5) | 4 (6.1) | 41 (21.5) | .01 |
| Mucosal bleeding | 54 (21.0) | 12 (18.2) | 42 (22.0) | .51 |
| Hepatomegaly | 13 (5.1) | 2 (3.0) | 11 (5.8) | .53 |
| Hematocrit change ≥20% & platelet nadir <50 x 10^9^/L | 53 (20.6) | 11 (16.7) | 42 (22.0) | .38 |
| **Laboratory parameters** |  |  |  |  |
| Platelet nadir x 10^9^/L  Median (IQR) | 28 (13 – 64) | 23 (13 – 60) | 29 (13 – 69) | .85 |
| White cells count – lowest x 10^9^/L  Median (IQR) | 2.8 (2.1 – 3.9) | 2.5 (1.9 – 3.7) | 2.8 (2.2 – 3.9) | .21 |
| Creatinine – highest x 10^9^/L  Median (IQR) | 92 (73 – 116) | 90 (70 – 114) | 93 (74 – 120) | .64 |
| **Treatment and outcome from hospitalization** | | | | |
| Platelet transfusion | 56 (21.8) | 14 (21.2) | 42 (22.0) | .90 |
| Length of stay – days  Median (IQR) | 6 (4 – 8) | 6 (5 – 7) | 6 (4 – 8) | .39 |
| ICU admission | 5 (1.9) | 0 (0.0) | 5 (2.6) | .33 |
| Death | 4 (1.6) | 0 (0.0) | 4 (2.1) | .58 |

Values are no. (%) unless stated otherwise.
